# G-protein coupled receptor 64 (GPR64) acts as a tumor suppressor in endometrial cancer

**DOI:** 10.1186/s12885-019-5998-1

**Published:** 2019-08-14

**Authors:** Jong Il Ahn, Jung-Yoon Yoo, Tae Hoon Kim, Young Im Kim, Russell R. Broaddus, Ji Yeon Ahn, Jeong Mook Lim, Jae-Wook Jeong

**Affiliations:** 1 Department of Agricultural Biotechnology, Seoul National University, Seoul, 08826 Republic of Korea; 2 Research Institute of Agriculture and Life Sciences, Seoul National University, Seoul, 08826 Republic of Korea; 3 Department of Biochemistry and Molecular Biology, Brain Korea 21 PLUS Project for Medical Sciences, Yonsei University College of Medicine, Seoul, 03722 Republic of Korea; 4 Department of Obstetrics and Gynecology & Reproductive Biology, College of Human Medicine, Michigan State University, 400 Monroe Avenue NW, Grand Rapids, MI 49503 USA; 5 Pathology, University of Texas M.D. Anderson Cancer Center, Houston, TX 77030 USA

**Keywords:** Endometrial cancer, Tumor suppressor, GPR64, Connexin 43

## Abstract

**Background:**

Endometrial cancer is the most common gynecological cancer. G-protein coupled receptor 64 (GPR64) belongs to a family of adhesion GPCRs and plays an important role in male fertility. However, the function of GPR64 has not been studied in endometrial cancer. Our objective is to investigate the role of GPR64 in endometrial cancer.

**Methods:**

We examined the levels of GPR64 in human endometrioid endometrial carcinoma by immunohistochemistry analysis. To determine a tumor suppressor role of GPR64 in endometrial cancer, we used a siRNA loss of function approach in human endometrial adenocarcinoma cell lines.

**Results:**

GPR64 levels were remarkably lower in 10 of 21 (47.62%) of endometrial carcinoma samples compared to control. Depletion of *GPR64* by siRNA transfection revealed an increase of colony formation ability, cell proliferation, cell migration, and invasion activity in Ishikawa and HEC1A cells. The expression of *Connexin 43* (*Cx43*), a member of the large family of gap junction proteins, was reduced through activation of AMP-activated protein kinase (AMPK) in Ishikawa cells with *GPR64*-deficicy.

**Conclusions:**

These results suggest that GPR64 plays an important tumor suppressor role in endometrial cancer.

**Electronic supplementary material:**

The online version of this article (10.1186/s12885-019-5998-1) contains supplementary material, which is available to authorized users.

## Background

Endometrial cancer is the most common gynecologic malignancy, with an estimated 63,230 new cases in 2018 [1]. The most common type of endometrial cancer is endometrioid adenocarcinoma, which originates from endometrial epithelial cells [2]. The development of endometrial hyperplasia, a proliferative process in the epithelium, is the abnormal thickening of the lining of the uterus due to an increase in the number of endometrial glands. It is a critical risk factor for endometrioid endometrial carcinoma [3]. Despite most cases being diagnosed in the early stages of endometrial cancer, a subset of these patients have poor outcomes and a high rate of recurrence and metastasis [4]. Recently, many studies have focused on targeted molecular therapies for controlling endometrial malignancies [5], however they are still insufficient. Therefore, it is important to identify molecular mechanisms involved in the development and progression of endometrial cancer.

G-protein coupled receptor 64 (GPR64) is a member of GPCR superfamily, which is crucial for male fertility [6–8]. *GPR64* was expressed in the proximal epididymis and efferent ductule regions with are responsible for spermatozoa maturation and rete testis fluid reabsorption [6–8]. In addition, expression of *GPR64* was found in fibroblast-like synovial cells in osteoarthritis [9]. The level of *GPR64* was higher in ewing sarcoma than other mesenchymal neoplasms, and GPR64 induces placental growth factor (PGF) and metalloproteinase (MMP1) expression [10]. Loss of *GPR64* in ewing sarcoma cell line leads to decreased PGF and MMP1 expression and reduced cellular growth with induced TRAIL dependent apoptosis [10]. Also, *GPR64* knock-down in an ewing sarcoma tumor model in immune deficient mice, reduced metastasis and invasiveness to the liver and lung [10]. GPR64 can activate G-proteins GS/Gq when over-expressed in xenopus melanophores [11]. Furthermore, *GPR64* was identified as a target gene of β-catenin/T-cell factor (TCF) in ovarian endometrioid adenocarcinoma [12]. However, the role of *GPR64* in endometrial cancer is unknown.

Connexin 43 (Cx43) is a member of the large family of gap junction proteins [13]. Gap junctions are intercellular plasma membrane proteins that provide for the exchange of ions and small molecules between adjacent cells [14]. Some studies have indicated that the Cx43 channel was localized at the plasma membrane, but not involved in Gap junction formation [15]. The Cx43 channel may regulate cell growth by transportation of calcium ions or other ions between intracellular cytoplasm and the extracellular environment [15, 16]. Other studies suggest that Cx43 can regulate cell growth and death by direct interaction with regulated cell cycle proteins including cyclin A, cyclin D1, p21, and p27 [17, 18]. Dysregulation of gap junction intercellular communication was to linked several human diseases such as cancer, cardiac ischemia, Charcot-Marie-Tooth (CMT), and Visceroatrial Heterotaxia Syndrom (VAH) [19, 20]. Cx43 is ubiquitously expressed in human tissues and controls cell growth and differentiation via multiple mechanisms. Attenuation of Cx43 is frequently observed in cancers, resulting in loss of gap junctional intercellular communication [21, 22]. Activation of Cx43 in cancer cells derived from various tissue types has been shown to result in restoration of normal cell growth and differentiation [23]. Small interfering RNA (siRNA)-mediated knockdown of *Cx43* results in a more aggressive growth of breast cancer cells [24]. Moreover, knock-out of *Cx43* in mice results in increased susceptibility to chemically induced lung adenomas [25]. There is an inverse correlation between Cx43 expression and tumor grade in endometrial cancer [26]. These observations suggest that Cx43 has a tumor suppression function and is a potential target in cancer therapy.

In this study, we examined the levels of GPR64 in human endometrioid endometrial carcinoma. To investigate the function of *GPR64* in endometrial cancer, we used *GPR64* siRNA in human endometrial cancer cell lines. Our results showed a new tumor suppressor role for *GPR64* in endometrial cancer.

## Methods

### Human endometrium samples

The human endometrioid endometrial carcinoma samples were obtained from The University of Texas MD Anderson Cancer Center. The control endometrial samples were obtained from hysterectomies (e.g., due to leiomyoma or a uterus prolapse). All patients with endometrial carcinoma underwent surgery. Twenty four controls and 21 endometrial cancer samples (not paired) were fixed in 10% buffered formalin prior to embedding in paraffin wax.

### Immunohistochemistry analysis

Immunohistochemistry analysis was performed as previously described [27]. Uterine cross sections from paraffin-embedded tissue were cut into 6 μm sections, mounted on saline-coated slides, deparaffinized and rehydrated in a graded alcohol series. For antigen retrieval, heat-induced epitope retrieval was performed using a pressure cooker with antigen unmasking solution (H^− 3300^; Vector Laboratories, Burlingame, CA) and then sections were pre-incubated with 10% normal rabbit serum in phosphate-buffered saline (PBS; pH 7.5) then incubated with anti-GPR64 (Sc-69,492; Santa Cruz Biotechnology, Dallas, TX) antibody in PBS supplemented with 10% normal rabbit serum overnight at 4 °C. The next day, sections were washed with PBS and incubated with secondary antibody conjugated to horseradish peroxidase (Vector Laboratories, Burlingame, CA) for 1 h at room temperature. Immunoreactivity was detected using diaminobenzidine (SK-4100; Vector Laboratories) then counterstained with hematoxylin and coverslipped with permount. Imunnostaining was analyzed using microscopy software from NIS Elements, Inc. (Nikon Instruments Inc., Melville, NY). A semi-quantitative grading system (H-score) was used to compare the immunohistochemical staining intensities.

### Cell culture and siRNA transfection

Human endometrial adenocarcinoma Ishikawa and HEC1A cells were maintained in phenol red–free DMEM/F12 medium (Gibco, Grand Island, NY) containing 0.1 mM sodium pyruvate (Gibco), 10% fetal bovine serum (FBS; Gibco), and 1% penicillin streptomycin (P/S; Gibco). Cells were cultured in monolayer at 37 °C in 5% CO_2_. *GPR64* siRNA was obtained from Dharmacon (Lafayette, CO), RNAi Technologies. Human *GPR64* siRNA was transfected using Lipofectamine 2000 reagent (Invitrogen Crop., Carlsbad, CA) prior to in vitro culture.

### Colony forming assay

After siRNA treatment, Ishikawa and HEC1A cells were seeded into 6-well plates at a density of 2 × 10^2^ cells per 2 ml cell culture medium and media was changed every 72 h for 14 days. Upon completion of culture, the plate wells were washed with PBS, fixed with 4% paraformaldehyde and permeabilized with 100% methanol. Colonies were stained with 1% crystal violet and images were captured via microscopy (Nikon Instruments Inc.) using software from NIS Elements, Inc.

### Cell proliferation assay

Cell proliferation was measured with a cell count kit-8 (Dojindo molecular technologies, Kumamoto, Japan) assay according to the manufacturer’s instructions. After siRNA treatment, 1 × 10^4^ cells were seeded into 24-well plate and cell proliferation was documented every 24 h for 10 days.

### Wound healing assay

Cell migration was measured by a wound healing assay. 1 × 10^5^ Ishikawa and HEC1A cells were seeded into 12-well plate and human *GPR64* siRNA was transfected into cells. After siRNA treatment, cells were incubated for 24 h. After 24 h, a pipette tip was used to create a scratch through the cell monolayer and cells were maintained in growth medium at 37 °C in 5% CO_2_. Cell migration was measured after 48 h, via inverted microscopy (Nikon Instruments Inc.) and distance was determined by Image J software (National Institute of Health, USA). Cell migration rate was converted to a percentage that measured the area compared to directly after the scratch.

### Invasion assay

For the transwell invasion assay, Ishikawa and HEC1A cells treated with siRNA were plated in the top chamber of a matrigel-coated membrane (24-well insert; pore size, 8 μm; BD Biosciences) at a density of 2.5 × 10^5^ per 200 μl serum-free culture medium and culture medium with 10% serum was used as a chemoattractant in the lower chamber. The cells were incubated at 37 °C in 5% CO_2_ for 48 h and cells that did not invade through the pore were removed by a cotton swab. Cells on the lower surface of the membrane were fixed with 4% paraformaldehyde and permeabilized with 100% methanol. Cells were stained with 1% crystal violet, and images were captured via fluorescent microscopy (Nikon Instruments Inc.) using software from NIS Elements, Inc.

### Annexin V/PI assay

Apoptotosis was measured with a FITC Annexin V Apoptosis Detection Kit I (BD Pharmigen, San Diego, CA) assay according to the manufacturer’s instructions. After siRNA treatment, 1 × 10^6^ cells were washed twice with cold PBS and resuspend cells in 1X binding buffer. 100 ul of the solution with 1 × 10^5^ cells was transfered to a 5 ml culture tube and added 5 ul of FITC Annexin V and 5 ul PI. The cells were gently vortexed and incubated at room temperature in the dark. After 15 min, 400 ul of 1X binding buffer was added to culture tube and analyzed by Flow cytometry (FACScalibur; Becton Dickinson, San Jose, CA). The data was analyzed using BD cell/Quest Pro software (Becton Dickinson).

### RNA isolation and quantitative RT-qPCR

Total RNA was isolated using the RNeasy total RNA isolation kit (Qiagen, Valencia, CA) according to the manufacturer’s instructions. As a template for quantitative RT-qPCR, cDNAs were synthesized using quantitative PCR random hexamers and MMLV Reverse Transcriptase (Invitrogen Crop.). The expression of *Cx43* was quantified by real-time PCR using a CFX96 Real-time Detection System (Bio-Rad Laboratories, Hercules, CA) and iQ™ SYBR Green Supermix (Bio-Rad Laboratories). *RPL7* expression was included in each treatment group for normalization. The sequences of the primers used for *GPR64* were 5′-CTGCAGGATCCCATTGTCTG-3′ and 5′-TGAAAGGGGTTGAATCTCCC-3′, for *Cx43* were 5′-ATGAGCAGTCTGCCTTTCGT-3′ and 5′-TCTGCTTCAAGTGCATGTCC-3′, and for *RPL7* were 5′-AAGAAGCGAATTGCTTTGAC-3′ and 5′-CAAATCCTCCATGCAGATGA-3′.

### Western blot analysis

Western blot analyses were performed as described previously [27]. Briefly, twenty five micrograms of protein lysates were electrophoresed via SDS-PAGE and transferred onto polyvinylidene difluoride membrane (Millipore Corp., Bedford, MA). The membrane was blocked with Casein (0.5% v/v) prior to exposure to anti-GPR64 (sc-69,492 Santa Cruz Biotechnology), anti-phospho-AMPK (Thr 172, #5235; Cell Signaling, Danvers, MA), anti-AMPK (#2532, Cell Signaling), anti-Cx43 (#3512, Cell Signaling), and anti-β-actin (SC-47778; Santa Cruz Biotechnology) antibodies. Immunoreactivity was visualized by incubation with a horseradish peroxidase-linked secondary antibody followed by exposure to electrochemiluminescence reagents (ECL) according to the manufacturer’s instructions (GE Healthcare Biosciences, Piscataway, NJ).

### Immunofluorescence

Immunofluorescence was performed as described previously [27]. Ishikawa cells were grown on glass coverslips and transfected with *GPR64* siRNA. Upon completion of growth, coverslips were washed with PBS, fixed with 4% paraformaldehyde and permeabilized with 0.1% of Triton X-100 (Sigma-Aldrich, St. Louis, MO). After further washing, Ishikawa cells were exposed to anti-GPR64 (sc-69,492; Santa Cruz Biotechnology) and anti-Cx43 (#3512; Cell Signaling) antibodes overnight at 4 °C and secondary antibodes for 2 h at room temperature. Washed coverslips were then mounted onto microscope slides with a DAPI-impregnated mounting media (Vector Laboratories) to enable nuclear visualization, and images were captured via a Zeiss LSM700 confocal microscope (Carl Zeiss microImaging GmBH, Jena, Germany) using ZEN 2009 software (Carl Zeiss microImaging).

### Statistical analysis

Cell experiments were measured in triplicate and averaged to achieve a single value for each combination of treatment, time point, and cell line. Statistical analyses were performed using one-way ANOVA analysis, Tukey’s post hoc multiple range test or Student’s t-tests using the Instat package from GraphPad (San Diego, CA). *p* < 0.05 was considered statistically significant.

## Results

### The levels of GPR64 are altered in a subset of endometrial cancer

**Fig. 1:**
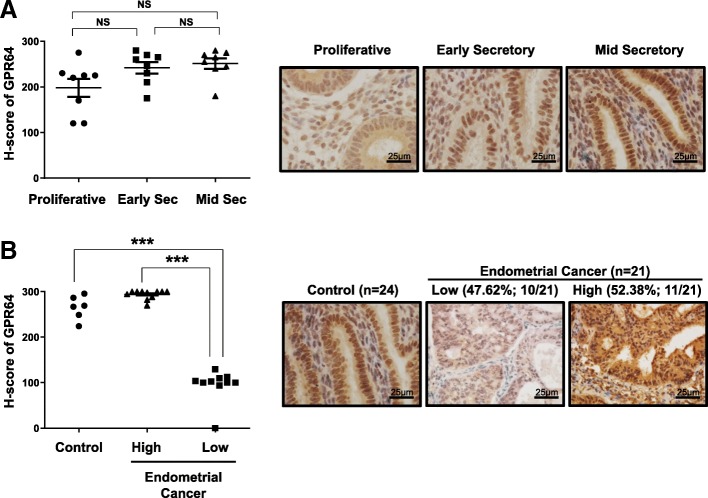
Levels of GPR64 in human endometrial cancer. **a** Representative immunohistochemistry andsemiquantitative analysis for GPR64 expression in control endometrium from women in proliferative, early, and mid-secretory phases. **b** Representative immunohistochemistry and semiquantitative analysis for GPR64 expression in endometrial cancer tissues. The semiquantitative analysis of immunohistochemistry analysis was calculated by H-score among 24 control endometrium and 21 endometrial cancer tissues. High level of GPR64 was observed in 100% control endometrium (6/6) and 52.38% endometrial cancer (11/21), and low level was observed in 47.62% endometrial cancer (10/21). ***, *p* < 0.001

To determine the levels of GPR64 in endometrial cancer, we performed immunohistochemical analysis using tissue from 24 controls and 21 endometrioid endometrial carcinoma samples. In control endometrium, GPR64 proteins were strongly detected in the nucleus, cytosol and apical membranes of stromal and epithelial cells of endometrium from the proliferative phase and secretory phases in women and the levels of GPR64 proteins were not significantly changed during the menstrual cycle (Fig. 1a). Interestingly, the levels of GPR64 were significantly lower in 47.62% (10/21) of endometrial cancer tissue compared with controls (*p* < 0.001). However, levels of GRP64 were unchanged in 52.38% (11/21) of endometrial cancer samples (Fig. 1b; *p* = 0.0841). Epididymal tissue was included for the immunohistochemistry of GPR64 as a positive control [28, 29]. The expression of GPR64 was detected in apical membranes as well as in some nuclei of epididymal duct epithelial cells (Additional file 1: Figure S1). These results suggest that GPR64 may play a tumor suppressor role in certain cases of endometrial cancer.

### Attenuation of *GPR64* increases colony forming ability and cell proliferation

**Fig. 2:**
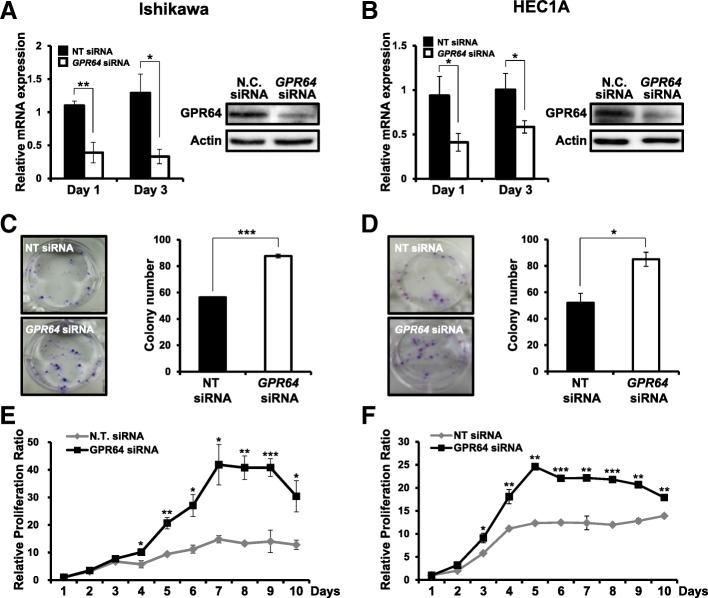
Effect of *GPR64* loss on cell growth in human endometrial cancer cells*.* **a** and **b** The expression of *GPR64* mRNA and protein in Ishikawa (**a**) and HEC1A (**b**) cells transfected with non-targeting pool (NT) siRNA or *GPR64* siRNA was examined by RT-qPCR and Western blot analysis, respectively. **c** and **d** Colony formation assay of Ishikawa (**c**) and HEC1A (**d**) cells transfected with NT siRNA or *GPR64* siRNA. Samples from each treatment were transferred to flat-bottomed 24-well plates and incubated. Cells were fixed and stained with crystal violet. The average colony formation number was quantified with crystal violet stained cells. **e** and **f** Cell proliferation assay of Ishikawa (**e**) and HEC1A (**f**) cells transfected with NT siRNA or *GPR64* siRNA. The results represent the mean ± SEM. *, *p* < 0.05; **, *p* < 0.01; and ***, *p* < 0.001

The continual unregulated proliferation of cells is essential to cancer development [30, 31]. To characterize the proliferative role of GPR64 in endometrial cancer, we performed a colony forming assay and a cell proliferation assay in Ishikawa and HEC1A cells transfected with *GPR64* siRNA. First, we confirmed the decrease of *GPR64* mRNA and protein levels by *GPR64* siRNA transfection compared to non-targeting pool siRNA by RT-qPCR and western blot analysis, respectively (Fig. 2 a and b). The colony formation assay enables us to determine the survival and proliferation of cells [32]. Colony formation ability was significantly increased in Ishikawa and HEC1A cells transfected with *GPR64* siRNA compared to control (Fig. 2 c and d). Furthermore, the proliferation levels of Ishikawa cells were significantly increased by *GPR64* siRNA transfection after 4 days (Fig. 2e). The proliferation of HEC1A cells was also significantly increased by *GPR64* siRNA transfection after 3 days (Fig. 2f). Next, we examined whether GPR64 regulates cell apoptosis using an Annexin V/PI assay. The ratio of apoptotic cells was not different between *GPR64* siRNA-transfected cells and non-targeting pool siRNA transfected cells of Ishikawa and HEC1A cells (Additional file 1: Figure S2). These results suggest that GPR64 suppresses epithelial proliferation of endometrial cancer cells.

### Attenuation of *GPR64* increases cell migration and invasion

**Fig. 3:**
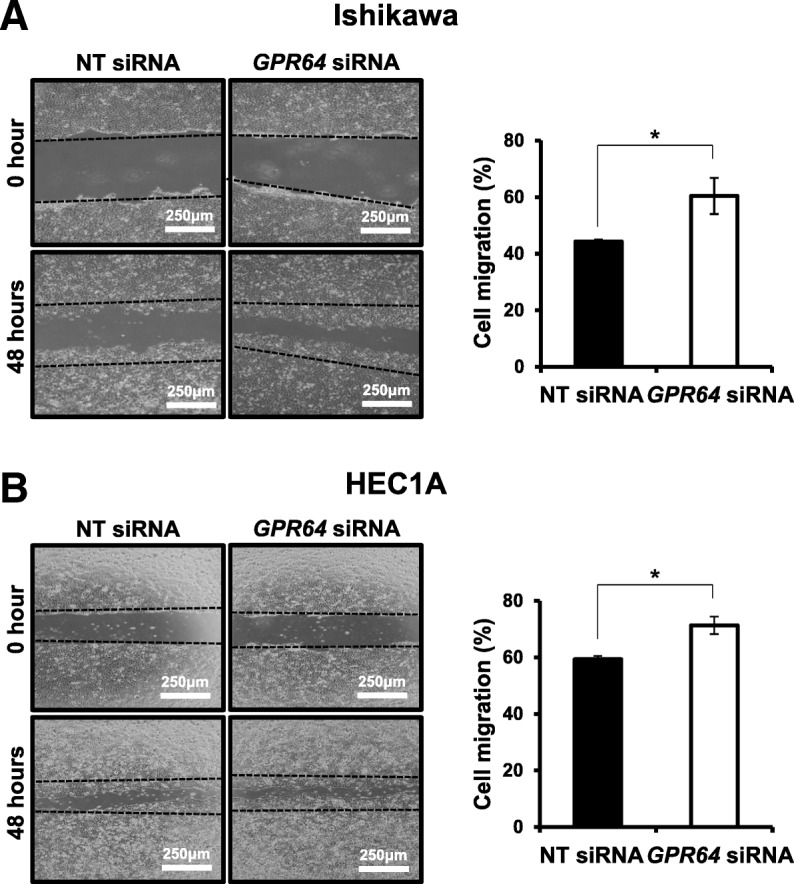
Ability of cell migration associated with *GPR64* expression in endometrial cancer*.* The wound healing assay was performed in Ishikawa (**a**) and HEC1A (**b**) cells transfected with NT siRNA or *GPR64* siRNA. After scratch, cells were incubated for 48 h to determine the cell migration ability. Representative result of wound healing assay and quantification of cell migration by wound healing assay during in vitro culture. The results represent the mean ± SEM. *, *p* < 0.05

We examined the effect of GPR64 attenuation in cell migration and invasion. Our wound healing assay revealed that reduction of *GPR64* by RNA interference significantly increased the migration ability of Ishikawa and HEC1A cells (Fig. 3). The *GPR64-*deficient Ishikawa cells recovered the wound over 61% after 48 h, while control cells healed less than 44% of wound distance (Fig. 3a). The HEC1A cells with *GPR64* knockdown recovered the wound over 71% but the control cells healed only less than 59% of wound distance (Fig. 3b).

**Fig. 4:**
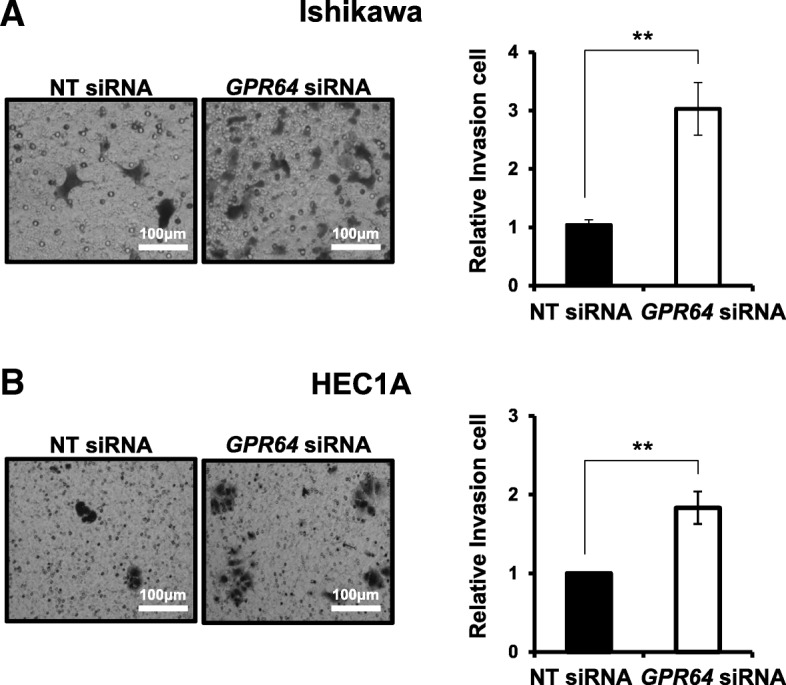
An increase of cell invasion by *GPR64* loss in endometrial cancer cells*.* Representative result of transwell invasion assays of Ishikawa (**a**) and HEC1A (**b**) cells transfected with NT siRNA or *GPR64* siRNA. Quantification of invasion through matrigel and transwell membrane in Ishikawa and HEC1A cells with or without *GPR64* siRNA treatment. The results represent the mean ± SEM. **, *p* < 0.01

Attenuation of *GPR64* exhibited a significantly higher infiltration rate in the transwell invasion assay of Ishikawa and HEC1A cells compared to siRNA controls. Invasion ability of *GPR64-*deficient Ishikawa cells was increased by more than three-times compared to control cells (Fig. 4a), and invasion ability of *GPR64* knockdown HEC1A cells was increased by more than two-times (Fig. 4b).

### Attenuation of GPR64 reduces the expression of Connexin 43 trough activation of AMPK

**Fig. 5:**
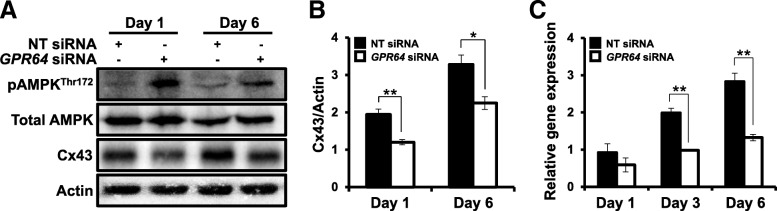
The reduction of Cx43 levels by *GPR64* loss through AMPK activation in Ishikawa cells. **a** The Western blot analysis of phospho-AMPK^Thr172^, Total AMPK, and Cx43 proteins in Ishikawa cells transfected with NT siRNA or *GPR64* siRNA on day 1 and day 6. Actin was used as sample-loading control. **b** Quantification of western blot analysis of Cx43. **c** The expression of *Cx43* gene during in vitro culture after *GPR64* siRNA transfection. *Cx43* was significantly decreased on day 3 and day 6 in cells transfected with *GPR64* siRNA. The results represent the mean ± SEM. *, *p* < 0.05; and **, *p* < 0.01

Activation of AMP-activated protein kinase (AMPK) plays a critical role in induction of EMT in multiple cancer cell types [37]. To determine whether AMPK activity is regulated by depletion of *GPR64*, we examined phosphorylation level of AMPK (Thr 172) in Ishikawa cells transfected with *GPR64* siRNA. The levels of phophorylated AMPK were increased by *GPR64* knock-down compared to control, however total AMPK levels were not different between control and *GPR64*-deficient cells (Fig. 5a).

Connexin proteins are frequently dysregulated in tumors, resulting in loss of gap junctional intercellular communication [21, 22]. Many tumors with decreased Cx43 expression exhibited dysfunctional gap junctional intercellular communication [19, 38, 39]. The expression and function of Cx43 have been correlated with carcinogenesis in endometrial cancer [26, 40]. Therefore, we examined the levels of *Cx43* proteins and mRNA in Ishikawa cells with *GPR64* knockdown using RT-qPCR and Western Blot analysis. Depletion of GPR64 significantly reduced the levels of Cx43 proteins on day 1 and day 6 compared with non-targeting pool siRNA by Western blot analysis (Fig. 5 a and b). The expression of *Cx43* mRNA was significantly reduced in the Ishikawa cells treated with *GPR64* siRNA on day 3 and day 6 compared with non-targeting pool siRNA (Fig. 5c).

**Fig. 6:**
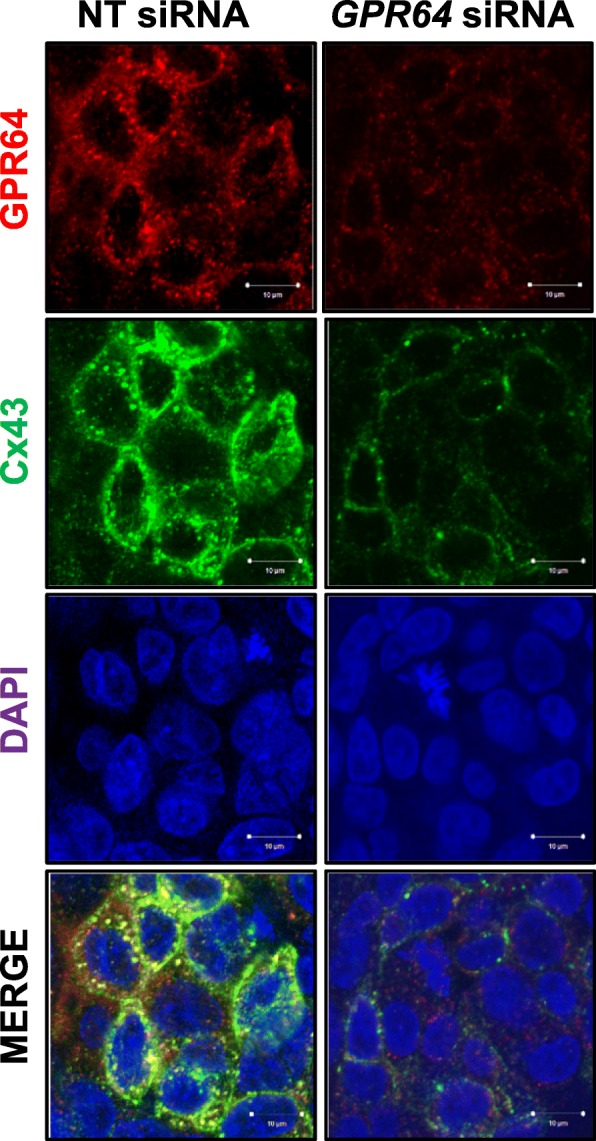
The colocalization of GPR64 with Cx43 in Ishikawa cells. The colocalization of GPR64 (red) and Cx43 (green) were analyzed in Ishikawa cells transfected with NT siRNA or *GPR64* siRNA by fluorescence microscopy. GPR64 overlaps with Cx43, but its colocalization was affected by reduction of *GPR64*. Nuclei were counterstained with DAPI staining

Next, we performed double immunofluorescence for GPR64 and Cx43 after *GPR64* knock-down (Fig. 6). GPR64 is colocalized with Cx43 in Ishikawa cells. However, the expression of Cx43 protein was remarkably reduced in *GPR64*-deficient cells by *GPR64* siRNA compared to non-targeting pool siRNA.

## Discussion

G protein-coupled receptors (GPCRs) are transmembrane receptors and play important roles in multiple biological processes. Aberrant expression of these receptors has been linked to cancer development and progression [43–45]. However, the molecular function of GPCRs is not known in endometrial cancer. The present study shows that the expression of GPR64 was distinctly lower in a subset of endometrioid endometrial carcinoma. These results suggest a tumor suppressor role of GPR64 in endometrial cancer.

Recently, Richter et al. identified that *GPR64* is specifically overexpressed in Ewing sarcoma (ES) but is also up-regulated in prostate, kidney, and lung carcinoma [10]. This study identified that suppression of *GPR64* in ES by siRNA, led to impaired colony formation, cell growth, and metastasis in Rag2^(−/−)^ γC^(−/−)^ mice. Moreover, suppression of GPR64 induced TRAIL mediated apoptosis and reduced PGF and MMP1 expression [10]. GPR64 is also involved in cell adhesion and migration through activation of serum response element (SRE) and nuclear factor kappa-light-chain-enhancer of activated B cells (NFκB) and its knockdown by siRNA in the highly motile breast cancer cell lines results in a reduction in cell adhesion and migration [46]. However, our results showed that depletion of *GPR64* increases the cell proliferation, migration, and invasion of endometrial cancer cells. These results suggest the role of GPR64 as a tumor suppressor in endometrial cancer. It remains possible that *GPR64* plays dual functions in different cancer conditions depending on the spatial and temporal distribution and abundance of different *GPR64* downstream targets and factors that regulate *GPR64*. Our results suggest a new role of GPR64 as a tumor suppressor in endometrial cancer, but further studies are needed to provide a conclusive answer.

Unfortunately, we could not characterize the subtype of endometrial cancers where GPR64 could be a tumor suppressor. The levels of GPR64 were not correlated with grade or stage of endometrial endometrioid adenocarcinoma which is the most frequently occurring endometrial cancer cell type [47]. Cancer classification in the clinic is primarily based on histological analysis in the proper clinical context. Although highly informative, histopathology can be hampered by limitation in its ability to distinguish subtypes of cancers and molecular signatures. Although we could not characterize the subtype of endometrial tumors based on GPR64 levels in this study, composite molecular profiling of tumor specimens is increasingly becoming recognized as an adjunct to traditional histopathology. Therefore, the molecular characterization of tumor types using GPR64 expression may help to identify additional objective tools to enhance the classification of endometrial cancer. However, there is a need to investigate a tumor suppressor role of GPR64 in specific subtypes of endometrial cancer.

Several adhesion GPCRs have been shown to be involved in cell adhesion and migration, hereby influencing tumor progression [46]. GPR26 is a potent regulator of energy homeostasis through controlling hypothalamic AMP-activated protein kinase (AMPK) activation [48]. AMPK inhibits Cx43 expression in bladder smooth muscle cells [36]. While total AMPK levels were not different between control and *GPR64*-deficient cells, the levels of phophorylated AMPK were increased by *GPR64* knock-down compared to control. AMPK is associated with cancer development as an important mediator in maintaining cellular energy homeostasis [33, 34], and its activity is increased by extracellular changes such as depletion of ATP, low glucose, and changes of NADPH levels [35]. Additionally, AMPK inhibits Cx43 expression in bladder smooth muscle cells [36]. Cx43 expression has been associated with a wide variety of cancers, including liver tumor, colon cancer, breast cancer, ovarian carcinoma, and endometrial cancer [41]. Its role in controlling cell motility and polarity contributes to cancer development and metastasis. Especially, Cx43 decreased with poor differentiation of endometrial cancer [26], and Cx43 is considered to be weakened in progression of carcinogenesis [40, 42]. Thus, our results suggest that GPR64 is a tumor suppressor in endometrial cancer by regulating Cx43 expression through regulation of AMPK activity.

## Conclusions

We found an attenuation of GPR64 expression in a subset of endometrial cancer. We demonstrated that depletion of *GPR64* induced tumorigenic potentials by promoting cell proliferation, migration, and invasion in endometrial cancer cells. GPR64 regulates the expression of *Cx43* and AMPK activity in endometrial cancer cells. These results suggest that GPR64 acts as a tumor suppressor in endometrial cancer.

## Additional file

Additional file 1: **Figure S1.** Expression of GPR64 in mouse epididymis. The immunohistochemistry for GPR64 was performed in mouse epididymis as a positive control. Immunohistochemical staining of mouse epididymis shows membranous and nuclear positivity in epididymal duct epithelial cells. **Figure S2.** Effect of GPR64 on cell apoptosis in human endometrial cancer cells. Annexin V/PI assay were performed in Ishikawa (A) and HEC1A (B) cells transfected with with non-targeting pool (NT) siRNA or GPR64 siRNA to determine the effect of GPR64 on cell apoptosis. The apoptotic cells were analyzed by Flow cytometry. No difference was found between there were no significant difference between NT siRNA and GPR64 siRNA treatments. (PPTX 330 kb)

## Data Availability

The raw data available upon reasonable request from the corresponding authors.

## Abbreviations

CMT: Charcot-Marie-Tooth
Cx43: Connexin 43
DAB: Diaminobenzidine
ES: Ewing sarcoma
ESR1: Estrogen receptor α
GPCR: G protein-coupled receptors
GPR64: G-protein coupled receptor 64
MMP: Metalloproteinase
PBS: Phosphate-buffered saline
PGF: Placental growth factor
PGR: Progesterone receptor
siRNA: Small interfering RNA
TCF: T-cell factor
VAH: Visceroatiral Heterotaxia Syndrom
