# Supplementary material for: G-protein coupled receptor 64 (GPR64) acts as a tumor suppressor in endometrial cancer
Source: BMC Cancer. 2019 Aug 14;19:810. doi: 10.1186/s12885-019-5998-1 (PMC6694613; doi:10.1186/s12885-019-5998-1)
Supplement: Supplementary file 1 — Figure S1. Expression of GPR64 in mouse epididymis. The immunohistochemistry for GPR64 was performed in mouse epididymis as a positive control. Immunohistochemical staining of mouse epididymis shows membranous and nuclear positivity in epididymal duct epithelial cells. Figure S2. Effect of GPR64 on cell apoptosis in human endometrial cancer cells. Annexin V/PI assay were performed in Ishikawa (A) and HEC1A (B) cells transfected with with non-targeting pool (NT) siRNA or GPR64 siRNA to determine the effect of GPR64 on cell apoptosis. The apoptotic cells were analyzed by Flow cytometry. No difference was found between there were no significant difference between NT siRNA and GPR64 siRNA treatments. (PPTX 330 kb) [file 12885_2019_5998_MOESM1_ESM.pptx]

## Slide 1
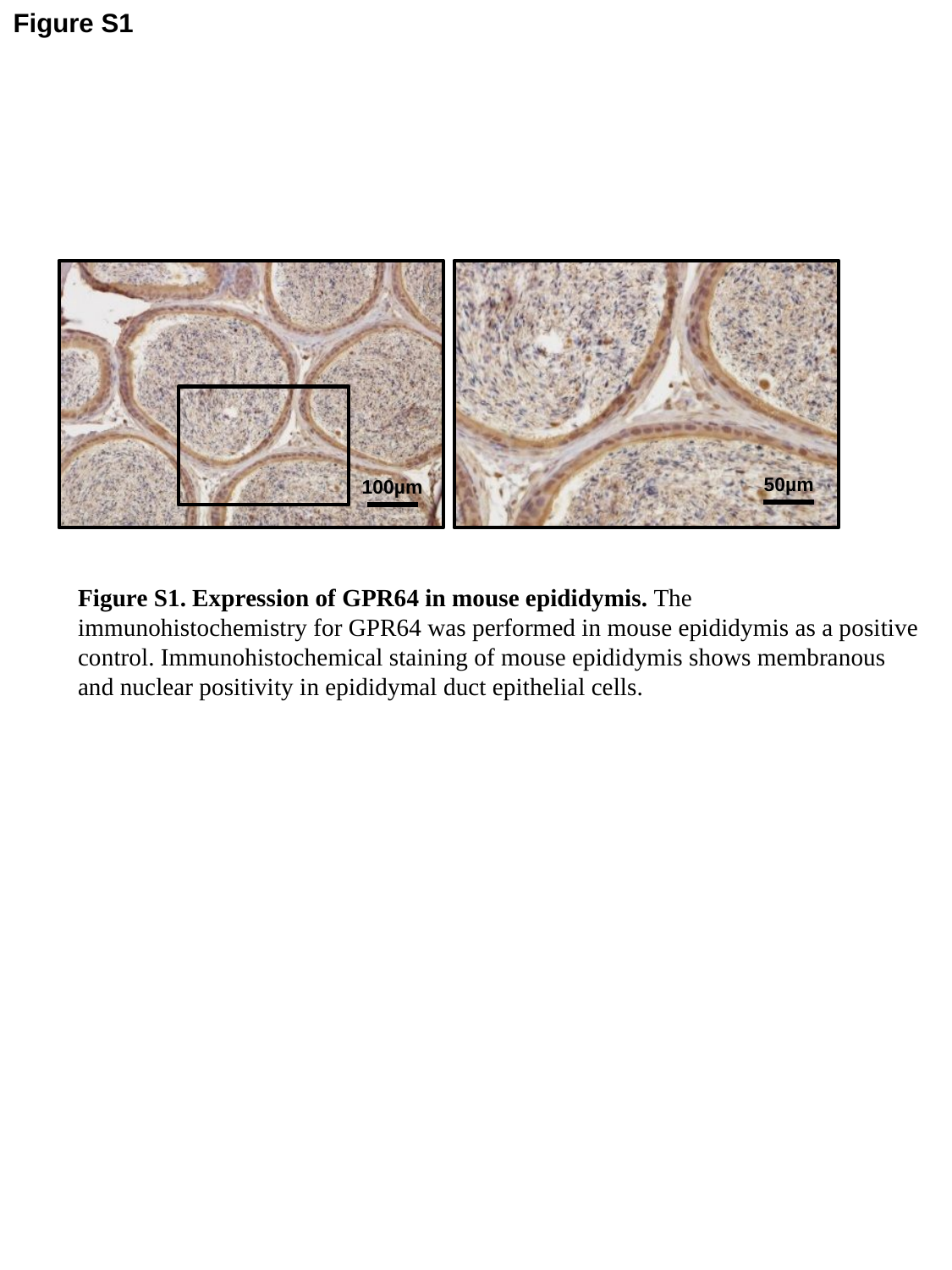

Figure S1
50µm
100µm
Figure S1. Expression of GPR64 in mouse epididymis. The immunohistochemistry for GPR64 was performed in mouse epididymis as a positive control. Immunohistochemical staining of mouse epididymis shows membranous and nuclear positivity in epididymal duct epithelial cells.

## Slide 2
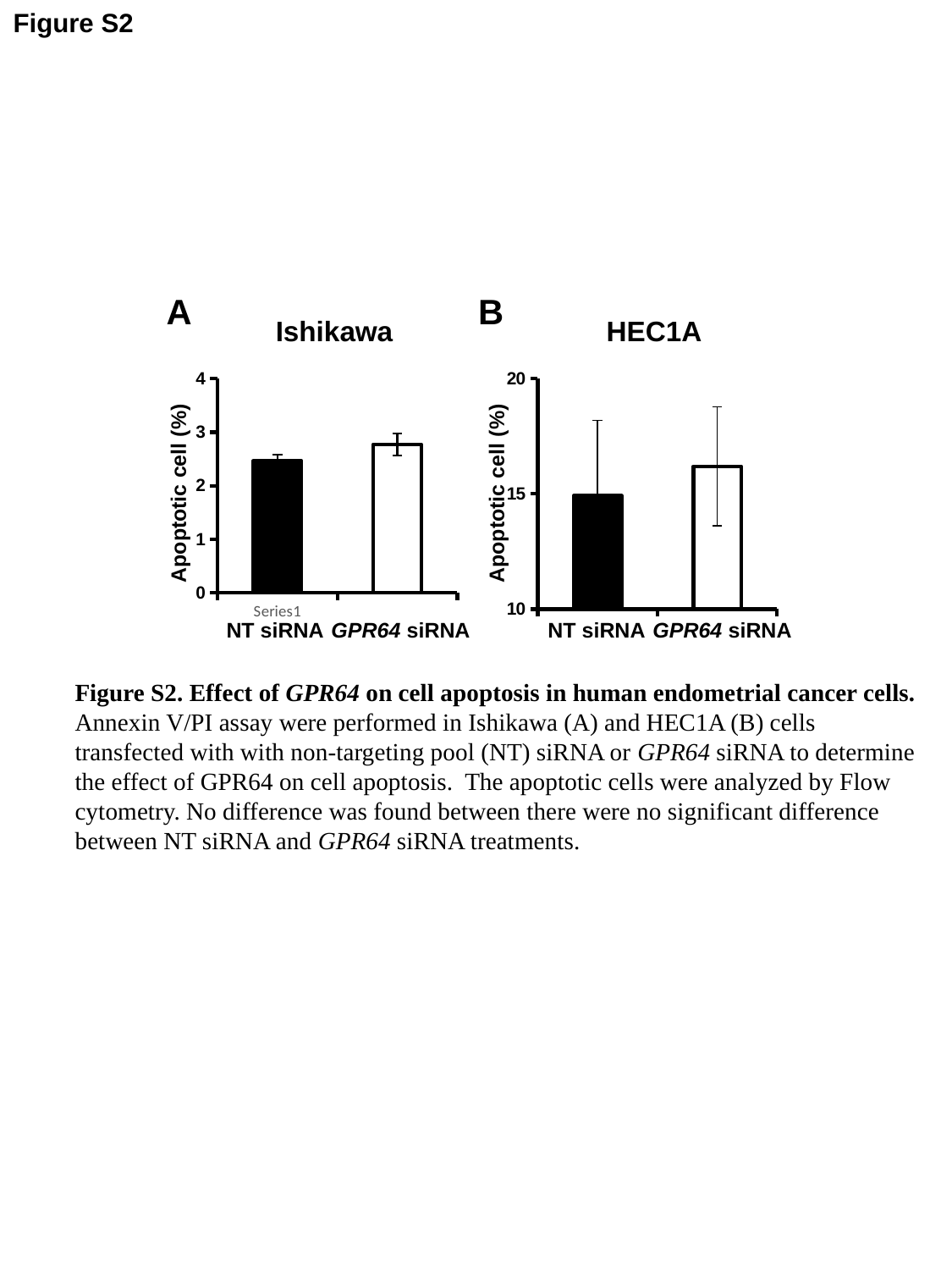

Figure S2
A
B
Ishikawa
HEC1A
### Chart
| Category | |
|---|---|
| | 14.913333333333334 |
| 1 | 16.19 |
### Chart
| Category | |
|---|---|
| | 2.4666666666666663 |
| | 2.776666666666667 |Apoptotic cell (%)
Apoptotic cell (%)
NT siRNA
GPR64 siRNA
NT siRNA
GPR64 siRNA
Figure S2. Effect of GPR64 on cell apoptosis in human endometrial cancer cells. Annexin V/PI assay were performed in Ishikawa (A) and HEC1A (B) cells transfected with with non-targeting pool (NT) siRNA or GPR64 siRNA to determine the effect of GPR64 on cell apoptosis. The apoptotic cells were analyzed by Flow cytometry. No difference was found between there were no significant difference between NT siRNA and GPR64 siRNA treatments.
